# Self-Reporting of Risk Pathways and Parameter Values for Foot-and-Mouth Disease in Slaughter Cattle from Alternative Production Systems by Kenyan and Ugandan Veterinarians

**DOI:** 10.3390/v13112112

**Published:** 2021-10-20

**Authors:** Julie Adamchick, Karl M. Rich, Andres M. Perez

**Affiliations:** 1 Department of Veterinary Population Medicine, College of Veterinary Medicine, University of Minnesota, Minneapolis, MN 55108, USA; aperez@umn.edu; 2 Department of Agricultural Economics, Ferguson College of Agriculture, Oklahoma State University, Stillwater, OK 74078, USA; karl.rich@okstate.edu

**Keywords:** foot-and-mouth disease, risk assessment, participatory methods, expert elicitation, Kenya, Uganda

## Abstract

Countries in which foot-and-mouth disease (FMD) is endemic may face bans on the export of FMD-susceptible livestock and products because of the associated risk for transmission of FMD virus. Risk assessment is an essential tool for demonstrating the fitness of one’s goods for the international marketplace and for improving animal health. However, it is difficult to obtain the necessary data for such risk assessments in many countries where FMD is present. This study bridged the gaps of traditional participatory and expert elicitation approaches by partnering with veterinarians from the National Veterinary Services of Kenya (*n* = 13) and Uganda (*n* = 10) enrolled in an extended capacity-building program to systematically collect rich, local knowledge in a format appropriate for formal quantitative analysis. Participants mapped risk pathways and quantified variables that determine the risk of infection among cattle at slaughter originating from each of four beef production systems in each country. Findings highlighted that risk processes differ between management systems, that disease and sale are not always independent events, and that events on the risk pathway are influenced by the actions and motivations of value chain actors. The results provide necessary information for evaluating the risk of FMD among cattle pre-harvest in Kenya and Uganda and provide a framework for similar evaluation in other endemic settings.

## 1. Introduction

Foot-and-mouth disease (FMD) is a highly contagious disease of livestock with massive global impact [1,2]. FMD costs billions of dollars annually due to endemic losses and outbreaks [3], and control measures such as vaccination, biosecurity, and stamping out when outbreaks occur are also costly [4,5]. Despite global efforts for FMD control [6,7], FMD remains endemic in many regions [8].

The Agreement on Sanitary and Phytosanitary Standards adopted by the World Trade Organization Member States [9] specifies that trade restrictions based on health hazards associated with the trade of goods should align with the guidance of international standard-setting bodies (the World Organization for Animal Health (OIE) in the case of transboundary animal diseases such as FMD). Actions should be based on the level of risk presented by the trade of goods, as evaluated through objective risk assessment. According to the principle of equivalence, countries are to recognize the actions taken by exporting partners according to the reduction in risk achieved rather than requiring a specific set of protocols (though actual practice is often murkier [10]). For this reason, risk assessments are an essential tool for demonstrating the fitness of one’s goods for the international marketplace as well as for understanding and improving animal and public health domestically [10,11].

Import risk assessment is typically used to inform risk management from the defensive standpoint of an importing country; it assesses how to reduce and mitigate the risk of importing a threatening bug or substance based on the probability and consequence of the event occurring. Countries that want to export are evaluated by potential importers using this approach and criteria. In order to export products that could potentially transmit FMD virus, countries have traditionally been required to demonstrate that FMD is not present in the region where cattle (or other source livestock or wildlife) are produced and processed. This requirement is costly, comes with tradeoffs and externalities, and has not been achievable for most of Africa [12,13]. Recent alternatives, which include disease-free compartments and commodity-based trade, encourage the examination of more nuanced, strategic approaches to the development of production and processing systems for export [14,15]. In this context, import risk assessment can be used by the exporting country to evaluate the risk (probability of FMD transmission) experienced by a potential importer under various production and processing scenarios. This analysis could then be used to lobby for access to external markets, or, if unacceptably high, to evaluate the potential value of interventions to reduce risk compared with net benefits from other markets with less stringent entry requirements.

However, in many countries where FMD is present, it is challenging to obtain the necessary data for such assessments, due in part to the small scale and non-standardized value chains that often operate with a mix of formal and informal processes and incomplete documentation of transactions [16]. In this study, we used a hybrid between participatory and expert elicitation techniques to overcome this gap. This novel approach, in which we partnered with local veterinary professionals to characterize risk pathways and parameter values, captured some of the richness and quality of data collected through participatory methods while maintaining the quantitative rigor required to utilize the data in formal risk assessment models.

There is a history in animal and public health fields of using participatory methods to overcome data scarcity challenges for epidemiological surveillance, research, and outreach [17,18]. A participatory approach to risk assessment has been developed and implemented for many studies of food safety in African markets and value chains [16,19,20] and more recently to qualitatively assess the risk of disease introduction and spread [21]. Efforts to marry value chain analysis with risk assessment have also attempted to connect participant knowledge of value chain dynamics with the assessment and management of risks related to animal and public health [22,23,24]. Participatory approaches promote both efficiency and impact by including populations that are affected by decisions made based on study findings [16]. Specifically relating to risk assessment, an advantage over conventional approaches is the chance to capture relevant aspects of human behavior as well as technical causal mechanisms contributing to risk pathways and probabilities [25]. However, a challenge encountered in participatory risk assessments is the need to generate robust evidence of the type that can be used for formal, quantitative risk assessment [16].

The elicitation of expert knowledge from subject matter experts is another approach utilized when data are scarce, unrepresentative, or inadequate to describe the process being studied [26,27]. “Expert” in this usage can refer to a person who can provide information about the question based on their experience with the subject matter of interest [28,29]. This approach has been used within veterinary science to estimate parameter values or prioritize risk factors [30,31,32,33,34,35]. However, when trying to collect information about local systems or informal pathways, a challenge is that those familiar with the subject may not have an academic understanding of the techniques being used. This can impede effective communication and impact the quality of the results if adequate training is not provided [26,27].

The hybrid approach employed here relied on partnership with Kenyan and Ugandan mid-career veterinary professionals who were enrolled in a capacity-building course that covered topics including international trade, transboundary diseases, and risk analysis. Their participation and contribution to the research generated credible data about the risk pathways and parameter values that can be used in a quantitative, probabilistic risk assessment to inform decisions about disease management based on local conditions and priorities. The richness of the data collected gave insight into causal relationships that can help inform appropriate model structure [36] and risk management strategies, including correlations between events in time and space and the influence of actors’ incentives on events that contribute to risk.

The objective of this study was to characterize the risk pathways for FMD among cattle at the time of slaughter in Kenya and Uganda through partnership with practicing veterinarians. That objective has been achieved through (a) describing the risk pathways and events; (b) defining the populations of cattle based on the production system of origin, which are expected to have distinct FMD risks associated with baseline conditions and processes; and (c) specifying parameter values to characterize events that require knowledge of the local sale and inspection processes (i.e., what happens between the farm and the abattoir). These results can be used to perform risk assessments, modeling exercises, and economic analyses regarding the expected value of investments based on empirical understanding of the local system. This framework may be used for similar analyses in other endemic settings, ultimately contributing to the analysis and design of targeted interventions for development of risk-based export markets. 

## 2. Materials and Methods

### 2.1. Risk Question

The question to be answered for each of four cattle production systems in two countries was: what is the risk that cattle sold for meats are slaughtered while infected with FMD? Mapping and quantifying that risk required system-specific knowledge of the events that occur prior to slaughter for cattle originating from local production systems. Expert knowledge was elicited from practicing veterinarians in Kenya and Uganda, separately, to describe the risk pathways, define populations of relevance, and quantify parameter values for key variables related to sale, transportation, and inspection of cattle.

### 2.2. Participant Selection

The subject-matter experts for this study were defined as veterinary professionals living and working in their respective countries (Kenya, Uganda) with at least two years of experience related to livestock production, and training in risk assessment for animal health and international trade. Experts were identified and contacted in the context of an online capacity-building course for mid-career Veterinary Service (VS) professionals (progressvet.umn.edu) in which they were trainees [37]. The procedures for recruitment and selection of participants in the training course differed between Kenya and Uganda. In Kenya, participants were nominated for the course by the national Directorate of Veterinary Services for the country; in Uganda, participants were self-selected with facilitation through Makerere University and the national Ministry of Agriculture, Animal Industry and Fisheries. The training was done in parallel for both countries (i.e., the instructors, materials, and procedures were the same, but there was no interaction between participants in Kenya with those in Uganda). At the time of the research study, which was five months into the program, they had completed five weeks of training on risk analysis applied to animal health and food safety. Thirteen Kenyan and ten Ugandan participants were in the program at the time when the study was conducted and comprised the pool of available subject matter experts. 

The elicitation activity—a guided exercise of building and quantifying a risk assessment model based on participant knowledge and experience—was part of the training program. This facilitated an approach that was a hybrid between traditional participatory and expert elicitation techniques. The participants—already experts on the subject matter of local cattle production and disease management systems—were recently trained as a cohort in topics related to the research question, methodology, and context. The context of the training program facilitated data collection through a prolonged, iterative process of gathering descriptive, qualitative information as well as quantitative parameter values, first at the level of individual responses followed by group discussion. The specific steps of data collection are outlined below. Further discussion of the duality of the training and research activities can be found elsewhere [37].

Participants were given the opportunity to opt in for their input during the training exercise to be used for research purposes, with the explanation that their choice would not have any impact on their standing or relationships in the training program. All individuals (*n* = 13 Kenya, *n* = 10 Uganda) chose to do so. The University of Minnesota Institutional Review Board for research involving human participants reviewed the study protocol and determined that it met the criteria for exemption from review.

### 2.3. Knowledge Elicitation and Integration

**Figure 1:**
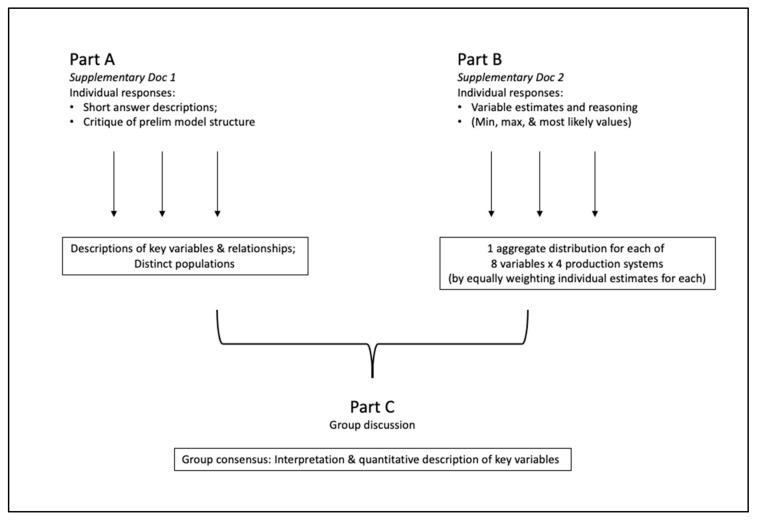
Schematic of the approach used. Parts A, B, and C were carried out separately for Kenya and for Uganda. Parts A and B were individual activities; the individual results were organized and aggregated to present to the group for discussion, revision, and final consensus in Part C.

The elicitation activities took place in three stages, referred to as Part A, Part B, and Part C, over a three-week period (see Figure 1). All activities were conducted separately for each country. The three stages comprised a variation of the Delphi method [38], an iterative process of eliciting individual responses and group discussion to reach consensus. Parts A and B were completed individually, helping to avoid dominance of any one opinion in the information gathered [28]. Part A comprised 18 open-ended, short answer questions. In Part B, participants provided quantitative estimates for parameter value distributions and were asked to only respond for the management systems with which they felt most comfortable. Part C was a group discussion to reach consensus regarding the values of key variables for all management systems; the aggregated values from Part B were provided as a starting point, and all participants were encouraged to comment on how they felt those distributions should be altered to best represent the range and distribution of values in each system. 

#### 2.3.1. Part A

The instructions, background material, and questionnaire for Part A were distributed in a similar manner to all previous assignments in the training program: via email as well as through an online learning platform (Canvas LMS, Instructure, Salt Lake City, UT, USA). Participants were able to fill out and return the questionnaire through either route. This was completed individually by each participant. The questionnaire consisted of four sections with 18 open-ended, short answer questions (Supplementary Materials File S1) interwoven with educational material related to the process of risk assessment and the role of expert opinion. This context-gathering phase, not often included in expert elicitation protocols, provided insight into correlational and causal relationships between events that otherwise may have been overlooked by the modeling team.

The first section contained seven questions about the sale, transportation, and inspection of cattle sold for slaughter in their country, including two questions that asked about possible correlations between events. In the second section, participants walked through the steps and logic of building a fault tree and event tree for a simple example risk model (the risk of sleeping through one’s alarm). They were then presented with preliminary outputs (a fault tree and event tree) of the same process applied to the combination of events that would lead to the outcome of cattle infected with FMD at the time of slaughter. They were asked whether the pathways presented made sense, whether they agreed, and whether they could identify any additional pathways. The preliminary model structure was built by the research team after a review of available literature. 

In the third section, participants were asked to consider how the risk could differ among animals originating from distinct production systems. Kenya and Uganda each have diverse cattle production systems including pastoralism, smallholder agropastoralism, and confined extensive and intensive farms. Beef cattle systems in each country have been classified by the FAO through a process that engaged key national stakeholders and synthesized sources of cattle distribution and production data [39,40]. The participants reviewed these classifications for their country, were asked for each of 11 variables whether they believed the value would be the same or different in each system and were asked if they would recommend a different way of dividing and identifying subpopulations. 

The fourth section was four open-ended questions reflecting on the processes that create and mitigate risk and the role of Veterinary Services. 

The anonymized individual responses were reviewed separately by three researchers, whose review was guided by the question: Do participant responses support, expand, or contradict the preliminary model structure (variables, relationships, and populations)? After reviewing the responses individually, the researchers discussed in which areas the responses indicated a consistent action to be taken and in which areas there was contradiction or ambiguity in their responses, requiring further clarification in later stages. As a result of that discussion, they had a list of aspects of the model structure to be accepted as is, modifications to the model structure, and additional information to be elicited during parts B and C. 

#### 2.3.2. Part B

Part B was a questionnaire intended to elicit quantitative and qualitative information about key parameter values for the risk model (Supplementary Materials File S2). The questionnaire was completed individually using web-based survey software (Qualtrics, Provo, UT, USA) by each participant. Instructions and background information was distributed through email and on Canvas. 

The questionnaire opened by presenting the subpopulations (production systems) for the cattle industry in the respective country, and participants were asked to select those for which they had experience and/or felt comfortable giving opinions about FMD risk and the farm-to-market process. For each production system they selected, participants were asked to estimate the minimum, maximum, and most likely value, and explain their reasoning, for 16 variables related to beef cattle production, sale, and inspection processes. They were instructed to reply “no answer” for any question if they did not feel they could provide a useful estimate.

Results were anonymized and aggregated for a selection of variables to be discussed by the whole group in part C. Variables were prioritized based on those which the population of veterinarians were well equipped to answer and for which there was little other information available. 

A noteworthy point of the elicitation process is that each participant provided both a point estimate (most likely value) and a distribution of uncertainty around that value (minimum and maximum possible). This is considered a better measure of uncertainty than simply taking the variability among several individuals’ point estimates [26]. Thus, our sample of 10 or 13 experts in each country yielded that many distinct distributions of the point estimate and uncertainty interval for each variable.

The distributions of each individual (specified as PERT distributions) were then combined into a single mixed distribution, weighting each one equally. This approach is outlined in risk assessment textbooks [41] and has been used elsewhere [42,43]. In our study, we used that mixed distribution as a starting place for group discussion, so that participants engaged with each others’ judgments of the range and most likely values to ultimately reach a consensus on the characteristics of the final appropriate distribution. This aligns with the recommended best practices for expert elicitation: including multiple experts, using a structured protocol for the phases of knowledge elicitation and aggregation, and providing the opportunity to interact and cross-examine reasoning within the group [26,29].

Answers were excluded from the aggregation if the respondent’s rationale indicated that they were estimating something other than what the question was asking. If the distributions and reasoning were similar across the four production systems, then they were merged into a single distribution; otherwise, they were kept distinct for each production system. Some variables were conceptually summarized or manipulated to form a new variable, related to but distinct from that which had been asked in the questionnaire, in order to be better formulated for input to a risk assessment model. More specific information about the aggregation approach for each variable is described below.

- Duration in days between sale and slaughter: direct mathematical aggregation was used for discussion.
- Probability of not commingling: The questionnaire asked about the probability of mixing with animals from other herds. The estimates given by each participant were subtracted from 1 to yield the probability of not mixing with animals from other herds. This complementary probability was aggregated into a composite distribution for each production system and presented for discussion in Part C.
- Number of animals mixed with, when commingling does occur: direct mathematical aggregation was used for discussion.
- Number and probability of inspections: The questionnaire asked participants to estimate the number of times an animal would be inspected for FMD and then to describe each inspection and to estimate certain attributes: the percent of animals that would be inspected, the sensitivity of the inspection to detect clinical FMD, and the percent of positive diagnoses that would be ignored or compromised. The number of inspections was summarized as a range of point values to initiate discussion in Part C. The probability of inspection was handled differently in each country based on the flow of conversation in Part C. In Uganda, the discussion about the number of inspections included the proportion of animals for which that number would be zero. In Kenya, the most likely value for the percentage of animals who undergo each inspection was used to calculate the complementary portion of animals that do not get each inspection, which was then combined across all inspections reported by an individual to calculate the proportion of animals that would not receive any inspection. These values were presented to the group in Part C as the starting point for discussion about the probability of bypassing inspection for animals from each production system.
- Effectiveness and type of inspections: For each inspection described by each participant, a distribution for “effectiveness” was calculated by multiplying the minimum, maximum, and most likely values of the sensitivity multiplied by the most likely value of the reporting rate (defined as the complement of the most likely value for the proportion of positive results ignored or compromised). The effectiveness therefore described the percentage of animals that would be detected and detained by each inspection. If no answer was given for the proportion of results ignored, the sensitivity was assumed to functionally represent the effectiveness. In each country, the inspections and corresponding effectiveness estimates were categorized into two types that emerged from the comments and descriptions in parts A and B. Because of this emergent nature, the definitions of type 1 and type 2 differed between countries based on the patterns in participant descriptions of inspections. The effectiveness distributions for all inspections of each type were aggregated as described above into a single composite distribution of effectiveness for each type of inspection in each country. By synthesizing responses in this way, the distributions for each type of inspection included a variety of specific inspection circumstances and contexts. One distinction that was not explicitly discussed was whether a region in which inspection occurred was currently under FMD-related quarantine measures. The inspection descriptions were used to quantify how frequently each type occurred at each location (checkpoints, farm, market, slaughter, or unspecified/blended) and what rate of inspections in each production system took place at each location. This was used to compute the relative frequency (weight) of type 1 and type 2 inspections for each production system.

#### 2.3.3. Part C

Part C was a structured group discussion held using a web conferencing system with the participants from each country (conducted separately for Kenya and Uganda). The purpose of the discussion was to reach group consensus on the distribution of values for key parameters for each production system.

For each variable to discuss, the facilitator presented a summary of the related question(s) asked in Parts A and B and representative comments pertaining to the interpretation and estimation of the variable. Then, the most likely, minimum, and maximum values specified by each respondent were presented, along with the density plot and summary statistics of the composite distribution. Participants were asked whether the summary presented was an accurate description of the distribution for a particular management system or for all management systems. If they agreed or disagreed, they were asked to provide their reasoning and, where relevant, to propose how they would modify the distribution presented. There was limited use of the poll function in the web conferencing system to gather participant opinions; most of the discussion occurred as direct conversation among participants and through the chat. To close the discussion of each variable, the facilitator summarized the consensus of the discussion up to that point and asked if there was any further comment. Once all participants expressed agreement or no objection, the discussion moved on to the next variable. 

There was one variable presented in Part C for which no information was collected in Part B (included after reviewing the responses to Part A). For this variable, participants were asked to estimate, out of 10 animals infected with FMD, how many would experience each of four distinct outcomes. Participants gave their answers in the chat (Uganda) or in a poll (Kenya) and then discussed with each other the reasons for variation in their responses. 

Responses to Part B were unevenly distributed among management systems in each country. Where there were no responses for a certain variable in a certain management system, the group was asked which system they thought it would be most similar to, and then they were asked to explain how they would modify the values for that similar system in order to represent the one for which no Part B data had been provided. 

The discussion was recorded and distributed via email so that participants who were unable to attend would be able to view it and were encouraged to submit any comments they had regarding the discussion. 

#### 2.3.4. Final Steps

For the few variables designated as important to quantify by VS opinion but without time to discuss in Part C, the individual descriptions in Part A and B were used to thematically classify the responses into relevant summary variables as described above, and the quantitative estimates were then mathematically aggregated to represent the composite distribution described by all of the responses for each variable. 

Following Part C, the modified distribution for each variable (based on group consensus or mathematical aggregation) was summarized as a probability distribution that could be used for input into a probabilistic risk assessment model. Values that were VS opinion of a probability were summarized as PERT distributions. Values that were estimates of a scalar (number of animals, inspections, or days) or test characteristics (inspection effectiveness) were summarized as a common probability distribution with appropriate theoretical characteristics. Where multiple distributions were considered, the one with the lowest AIC was chosen. Distributions were fit using maximum likelihood estimation (package “fitdistrplus” [44], R software version 4.0.2 [45]). 

The distributions were presented back to each group for final comment, along with the consensus of the discussion and reasons supporting that consensus. Each distribution was described with accessible summary statistics. The report was distributed to the participants via email, and they were asked to review it and respond via email or in a virtual forum with any questions or comments. 

## 3. Results

In Kenya, there were 12/13 responses to Part A, 13/13 responses to Part B, and 6/13 active participants in Part C. In Uganda, there were 10/10 responses to Part A, 10/10 responses to Part B, and 9/10 active participants in Part C.

The veterinarians in both Kenya and Uganda unanimously confirmed that there was value in evaluating risk separately for distinct cattle production systems. Most respondents (9/10 Uganda, 11/12 Kenya) indicated that the management systems presented were appropriate classifications of beef cattle production systems in their country. 

### 3.1. Pathways

#### 3.1.1. Additional Event Added to the Proposed Risk Pathways

Most participants (8/10 Uganda, 12/12 Kenya) concurred with the risk pathways presented in the preliminary model of Part A. Two individuals in Uganda and three individuals in Kenya proposed an additional event be included on the pathway to represent the inspector’s decision to appropriately report and act on an FMD-infected animal. “We assume the right action will be taken but that is not always the case”, explained one Kenyan response.

**Figure 2:**
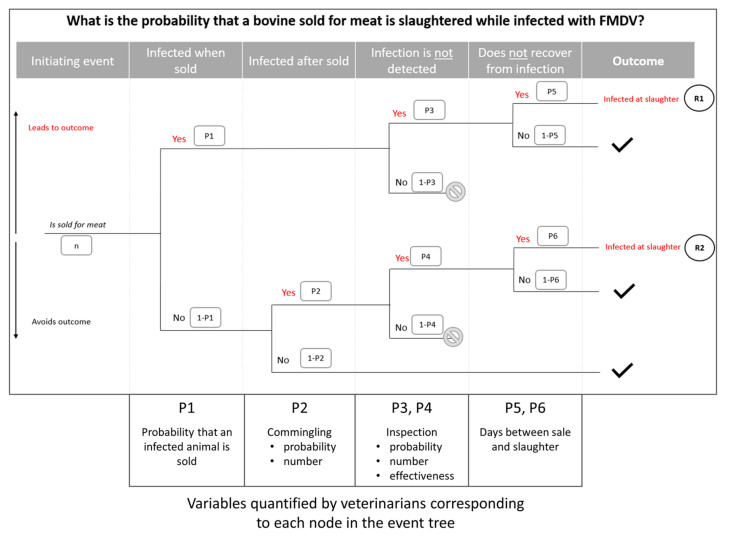
Event tree with risk pathways and variables characterized by veterinarians in Kenya and Uganda.

Following these responses, the event tree and risk pathways were updated similarly for each country. The event tree (Figure 2) was included in the final report back to the participants for review; it includes the steps from the preliminary model that participants supported and the additional step for the probability that appropriate action is taken by inspectors when an infection is suspected. There was no objection from any participant with the formulation of the resulting pathway.

#### 3.1.2. Correlations Exist between Events

Four Ugandan and three Kenyan participants indicated that points exist where an animal with FMD would be more likely to be sold for meat than an FMD-free animal. The Ugandan participants described that farmers at times want to dispose of animals that are sick, that farmers may sell animals when there is an outbreak in the area but quarantine is weakly enforced, and that during an outbreak farmers may want to dispose of affected animals to avoid losses. They also indicated that there may be temporal (seasonal) correlations between disease incidence and sales volume due to factors related to both demand (e.g., festivals) and supply (e.g., need for income at beginning of school year, decreased forage available during dry season). Kenyan responses described circumstances when farmers want to dispose of sick animals and traders to buy animals at a cheaper rate.

In contrast, three Kenyan and two Ugandan individuals indicated that there was no point at which an animal with FMD would be more likely to be sold for meat compared to a healthy animal. Several responses (six in Kenya and four in Uganda) discussed the possibility of selling FMD-infected cattle but did not address the question of correlation or comparison between sick and healthy animals.

### 3.2. Parameter Values

Participants estimated the minimum, maximum, and most likely value of variables for any/all production systems for which they felt comfortable responding. For Uganda, there were the following numbers of responses for each production system: Semi-intensive—7; Agropastoral—6; Ranching—2; Pastoral—1. For Kenya, there were the following numbers of responses for each production system: Pastoral—10; Agropastoral—3; Feedlot—1; Ranching—0.

Individual responses were aggregated into a composite distribution, which was presented and discussed with the cohort to reach a consensus on the characteristics of an appropriate distribution for each variable and each production system. The consensus, final parameters, and summary statistics for each are reported in Table A1 and Table A2 in Appendix A for Kenya and Uganda, respectively.

#### 3.2.1. Probability That an Infected Animal Is Sold While Infected

A discussion question was added to Part C following the responses about a possible correlation between the probability that an animal is infected with FMD and the probability that an animal was sold. The group was asked, out of 10 infected animals at random (throughout the year), how many would experience various outcomes including that the animal sold from the farm without reporting infection. In Uganda, the group consensus was that two to four out of every 10 infected animals are sold, for all production systems. The participants reasoned that it is hard for a farmer to report to the authorities that an animal is infected unless discovered by a professional because there is no form of compensation and that, when farmers realize there is disease in their region, they tend to sell animals to make sure their farms are empty. In Kenya, the group consensus was that two to three out of every 10 infected animals are sold on average across all production systems. 

#### 3.2.2. Duration of Time (Days) between Sale and Slaughter

The duration in days between when a cow leaves the herd and slaughter was described qualitatively in Part A, estimated in Part B, and discussed in Part C. The group consensus in Uganda was that the distribution for the duration of the process was similar for all production systems and that sources of variation, primarily the distance between origin and destination, could vary within any of the systems. They specified that this range does not include scenarios in which the purchased animals are held by a trader or butcher for extended lengths of time prior to slaughter. The Kenyan cohort concluded that the duration is different between production systems: pastoral and agropastoral systems had longer maximum durations and a larger variation, with pastoral having the longest most likely value (eight days) due to the distances the animals typically travel to reach the final destination. Feedlot and ranching systems had much shorter described durations, maxing out at two and three days, respectively, due to the shorter distance to travel and vertical integration in some systems.

#### 3.2.3. Commingling with Animals from Other Herds: Probability, Number

Situations in which commingling occurs were described qualitatively in Part A. In Part B, participants estimated the proportion of animals from each management system that do not commingle with animals from other herds before slaughter and then, for those which are exposed to animals from other herds, the number of animals with which they are mixed. In both countries, it was agreed that the probability of commingling would vary by management system, and the distribution for the number of animals mixed with when commingling does occur was the same for all cattle regardless of origin. The Ugandan group discussed that the probability of avoiding commingling was highest for animals from ranching systems (most likely value of 40%), and lowest (0%) for animals from pastoral systems. Participants commented on the general trend that in systems where farms have fewer animals, there would be more mixing on the way to market. In Kenya, individual and group discussions highlighted a distinction in the probability of avoiding commingling between systems that trek cattle to market on foot (identified as pastoral, agropastoral) and those that transport animals on trucks directly to a slaughterhouse premise (feedlot, ranching). This was attributed to the length of the journey, opportunities to congregate with other animals at markets or stops, and the number of animals sold at once from a single herd (e.g., enough to fill a truck with animals from the same origin). 

#### 3.2.4. Inspection: Probability, Number

Participants described inspection points and procedures from farm to slaughter. The responses from Uganda highlighted differences in the probability of inspection between systems based on the availability of veterinary services and the motivation of producers to maintain credibility and follow regulations. In the discussion in Part C, participants reinforced that it was not uncommon for animals from any system, and especially the three systems other than ranching, to completely bypass inspection before slaughter. They pointed to the current (at the time) movement restrictions in place in one district because of an FMD outbreak and that cattle were, regardless, being moved and slaughtered through unofficial channels. The consensus after some discussion was that the probability that an animal is never inspected (number of inspections = 0) was influenced most heavily by the destination for slaughter: if at designated slaughter points, they will be inspected; those that miss inspection are those going to undesignated slaughter points (“local slabs”). Animals from ranching systems were more likely than those from other systems to go to a designated slaughter facility and therefore had a lower likelihood of receiving 0 inspections. 

Five Kenyan participants indicated in Part A that they expected the probability of bypassing inspection completely (i.e., for whom the number of inspections is zero) to be higher among cattle from pastoral or agropastoral systems than those from feedlots and ranches. Individual estimates posited that 1% of animals originating from a feedlot were expected to bypass inspection completely, while up to 20% of agropastoral and 70% of pastoral cattle could potentially reach slaughter without being inspected. They reasoned that pastoral systems include vast areas that are poorly covered by all services including veterinary services, though others pointed out that inspection and permits are mandatory for all animals transported from one point to another. Others commented that buyers are motivated to perform their own inspections and check animals for indications of poor health that may cause losses; they want to “avoid being duped.” In the group discussion, the Kenyan cohort concluded that the probability of bypassing inspection differs by management system, with the lowest probabilities for animals from feedlot and ranching systems and a higher frequency and broader distribution of occurrence for animals from agropastoral and pastoral systems. The broad range for pastoral and agropastoral systems included acknowledgment that some of those inspections would be performed by community health workers or other non-veterinarians. The group emphasized that the percentage would be very low for cattle sourced from feedlots, since the animals and systems are closely monitored. 

#### 3.2.5. Inspection: Effectiveness

Participants described potential inspection points and estimated the sensitivity as well as non-reporting rate for each.

Among Ugandan responses, there were 27 inspection points described in total (2 pastoral, 10 agropastoral, 4 ranching, 11 semi-intensive). The inspection descriptions and distributions were similar for all production systems, so they were aggregated into a single distribution of effectiveness. Both the descriptions and the distribution indicated there were multiple “types” of inspections being lumped together. Based on the descriptions, inspections were categorized into two types:

- Rigorous (type 1): qualified and experienced personnel conducting exams, thorough inspection, “clinical signs are very clear”;
- Lesser (type 2): Any of the following: less qualified personnel (different incentives/stakes), less experienced, or less thorough (rushed, poor conditions/facilities, etc.), “clinical signs not always distinctive”.

There were 15 inspection points classified as type 1. All 15 individual distributions had a most likely value of 0.70 or greater, and the median value for the combined distribution was 0.83. There were 12 inspections classified as type 2. Ten of the twelve had a most likely value of 60 or lower, and the median value for the combined distribution was 0.52. Five of the inspections included an estimate for the probability that a positive result was ignored or compromised, with the most likely value ranging from 0.01 to 0.05 with a median of 0.02.

Kenyan responses described 21 inspection points (2 feedlot, 6 agropastoral, 13 pastoral). Descriptions and reasoning for each inspection delineated two types based on the occasion for inspection and who was performing it.

- Formal (type 1): any inspection performed by veterinary or animal health professionals before movement to the next stage (e.g., a movement permit before transportation or antemortem inspection before slaughter). Results from formal inspections were unlikely, but possible in some instances, to be ignored or falsified;
- Informal (type 2): performed by a trader, owner, butcher, or other middleman before the sale takes place. Results from these inspections were more likely to be compromised or ignored in the opinion of some VS members.

There were 16 inspections classified as type 1. Fifty percent of type 1 inspections had a most likely value of effectiveness greater than 0.90, and the median value for the combined distribution was 0.71. There were five type 2 inspections, four of which had a most likely value of 0.60 or lower. All inspections for feedlot cattle were described to be formal inspections; this was attributed to ranching systems as well based on the descriptions in Part A. Nine of the inspections included an estimate for the probability that a positive result was ignored or compromised, with the most likely value ranging from 0.0003 to 0.9 and a median of 0.2.

## 4. Discussion

In this study, we partnered with veterinarians in Kenya and Uganda to characterize the pathways and events leading to FMD infection at the time of slaughter among distinct populations of cattle in Kenya and Uganda. We then estimated values for key variables along those pathways from farm to slaughter based on the expert knowledge of veterinarians in each country. We found that risk processes differ between management systems, that disease and sale are not always independent events, and that events on the risk pathway are influenced by the actions and motivations of value chain actors including the decision of inspectors to report or to ignore an animal they suspect to be positive for FMD. The findings provide necessary information for evaluating the risk of infection among cattle at the time of slaughter in Kenya and Uganda and provide a framework for similar evaluation in other endemic settings. This knowledge can be used to guide exporter decisions for the development of risk-based export markets. A similar approach may be used to collect data to inform risk-based approaches to support the trade of various commodities from many geographies relating to FMD or other transboundary animal diseases.

The results describe differences in the risk processes among animals from distinct production systems. In the Kenyan systems, a trend emerged with clear delineation between pastoral/agropastoral and ranching/feedlot systems for several variables including the time from farm to slaughter, the probability of commingling en route, and the probability of bypassing inspection. The clustering of production systems whose characteristics extend beyond the farm gate is supported by other studies of Kenyan value chains [46,47]. The delineation between types of systems for factors contributing to the risk of acquiring a new infection en route to slaughter (in particular the probability of commingling with cattle from other herds) may be a strong indicator of which systems have the capacity to most easily adapt to an approach that involves direct transport and completely eliminates opportunities for exposure to other animals. Distinctions in management practices that occur on-farm were not characterized in this study but could be considered for future analysis: for example, routine vaccination against FMD. The impact of this variable would be partially captured by disease prevalence estimates but could also affect outcomes later in the pathway such as the probability of detection due to the rate of subclinically infected animals. A quantitative model could employ sensitivity analysis to explore how sensitive the overall risk is to the probability of displaying clinical signs and to other factors associated with vaccination (e.g., transmission probability from infected animals).

The events of FMD infection and sale for slaughter are not always independent for cattle in Kenya and Uganda due to both causal and correlational factors described by veterinarians in each country. Temporal and spatial patterns in FMD incidence, animal movements, and meat supply and demand have been described elsewhere [46,48,49]. Three participants (two Kenyan, one Ugandan) described the beginning of the school year as another time when producers would be more likely to sell cattle because of the need to pay school fees. The seasonal patterns may cause correlations between disease incidence and likelihood of being sold such that the prevalence of FMD infection among animals sold is different from the disease prevalence in a herd or region when expressed as the annual average. Furthermore, responses indicated that the presence of FMD in a region, herd, or individual could impact the probability of sale through various mechanisms. Other sources have reported the practice of informal sales continuing in Uganda even when an FMD quarantine is in place [50,51] and that the implementation of formal control measures such as ring vaccination may not be implemented for weeks after the initial outbreak event [49,52].

If disease and sale are not independent of one another, it may not be appropriate for a risk assessment to assume that animals sold are chosen at random from a herd and therefore that the risk of infection for that animal is represented by the average risk of infection for any animal in the herd. This assumption is common in risk assessments performed in the field of animal health and is often appropriate for a particular question and context [53,54,55]. However, for risk assessments examining the movement or sale of animals in endemic environments [56,57,58,59], our findings suggest it would be judicious to characterize the relationship between sale and disease of cattle in the population of study and to interpret the results of the risk assessment accordingly. While there are many studies on livestock marketing [60,61,62] and many on FMD epidemiology [63,64], this gap highlights the opportunity for further research on the relationships and mechanisms connecting the two. Such an insight would contribute to a fuller understanding and more accurate assessment of risk among animals originating from distinct production systems in FMD-endemic areas. 

The decisions of value chain actors influence the ultimate risk level in the product. The role of such decisions was highlighted and exemplified by the suggestion, made independently by multiple individuals in each country, to include a variable that accounts for the action taken by the inspector after diagnosing an animal as positive or suspect for FMD. Corruption is a barrier to health care access in many countries [65], has been described during regulatory inspection of pharmacies in Uganda [66], and may be incentivized among livestock producers by quarantine measures and disease control policies that restrict access to markets [67]. Actor motivations and incentives to make a decision in a given situation should be considered when building the structure of a model for risk assessment or economic analysis, especially where there may be feedback loops that could qualitatively change the conclusions of an analysis [10,68,69]. Utilizing risk analyses for identifying opportunities and designing effective policies requires understanding and acknowledging the role of motivation and incentives [70], including how they will change over time and the expected changes in actions taken [71,72]. In this particular case, it should be acknowledged that some participants may have been reluctant to provide quantitative estimates for the occurrence of compromised inspection results or to have open discussion with the group about this topic. This could have resulted in an overestimation of “effectiveness”, considering that the sensitivity was assumed to incorporate the impact of compromised results when not explicitly stated.

The approach used here, a partnership with local professionals in a hybrid between participatory and expert elicitation techniques, is a novel contribution to import risk assessments, particularly in disease-endemic and data-scarce settings. Participatory mapping and characterization of the risk pathways and value chains gathered valuable information about the processes and relationships at work, as described above. By utilizing local veterinary expertise to guide the model structure, this approach elicited information to help achieve the purpose of evaluating risk from the perspective of the importer but for the purposes of the exporter—giving insight into causal relationships to help inform an appropriate model structure [36] and risk management strategies [73]. Earlier uses of participatory methods for risk assessment have faced the challenges of “coupling” the beliefs of participating stakeholders with technical contributors when they differ [25]. In this case, since we considered our participants to be subject matter experts, we deferred to their beliefs in the realm of information discussed, and the procedures were in fact designed so that participants would update and improve the research team’s preliminary drafts and impressions of the systems obtained from generic or external sources. Robust and systematic procedures for training, eliciting, and reviewing participant knowledge helped to minimize bias and generate risk pathways and parameter estimates suitable for use in a formal model. At the same time, it is the hope and intention that the veterinarians and their communities also benefited from their involvement [37]. As professionals who are invested in improving animal health and livestock systems, their planning and decisions impact the outcome being discussed. It is reasonable to expect that the participatory exercise of mapping and interrogating the system, risk factors, and relationships from many professional viewpoints contributed to an updated understanding of their own role related to FMD and trade [74].

The results of this risk assessment and others can be used to develop risk-based approaches for FMD control at both the country and regional levels. Considering that risk dynamics, including many of the factors characterized here, change over time, it is best that each country’s veterinary services take on the task of regular risk assessment to guide risk management activities. The challenge of data scarcity can be addressed through the use of regular VS activities, e.g., data on health certificates, market throughput, and veterinary inspection reports. The results presented should be combined with other data related to herd management and sales, FMD prevalence, and disease transmission to complete a quantitative risk assessment. This should include epidemiological data that are representative of production systems’ distribution across each country to account for regional variations in disease occurrence. Risk-based approaches for FMD control, informed by the use of risk modeling and risk analysis, would aid early detection and response to disease outbreaks. Currently, reporting of outbreaks is often delayed or does not occur; there is limited surveillance for disease. Official outbreak reports differ by source [4] and appear to underestimate the occurrence of disease when compared to seroprevalence estimates. Common strategies for disease control include movement controls and ring vaccination in the face of outbreaks. The effectiveness of such measures are challenged by lack of resources for robust responses, delays in obtaining and delivering an effective response (median reported times between recognition of outbreak and deployment of vaccines in Uganda of 25 and 52 days in two separate surveys [49,52]) and underreporting of disease events, compounded by difficulty delivering veterinary service to remote areas. Vaccination is used primarily as a reactive rather than proactive measure of disease control in Uganda [49,52]; routine preventive vaccination has increased in Kenya in recent years [4].

The risk pathways reported here need to be coupled with activities at the slaughterhouse to characterize risk of transmission associated with the final product [75]. Handling practices at the level of the abattoir differ between facilities, based on their target markets. Most beef in Uganda and Kenya goes to domestic consumption and is slaughtered at some level of local slab or abattoir. Studies of abattoirs and butcheries in Uganda have demonstrated unacceptably high microbial contamination in meat samples as well as poor hygiene standards and beef handling practices [76]. Studies of meat handlers at five small and medium slaughterhouses in Nairobi likewise reported poor hygiene practices and microbial profiles that could facilitate cross-contamination of meat [77,78]. Meat inspection is often performed by a Public Health Officer, with greater emphasis on zoonoses and foodborne pathogens than trade sensitive diseases such as FMD. Larger processing companies with their own abattoir may be expected to have superior sanitary processes due to company standards but handle a small portion of the beef supply (11–13% in Nairobi [46]). There are approximately five abattoirs or companies in each country that export meat and offal to other countries in the region [79]. In addition to hygiene practices, it would be critical to describe the actions taken when an animal or carcass is identified to have FMD in order to understand the implications for risk in associated animals or meat products.

The primary limitations of this study are related to the use of expert knowledge as a surrogate for empirical data [80]. Rigorous methods must be utilized to obtain accurate and reproducible study results in the face of motivational, behavioral, and cognitive biases [81]. This study included many of the core tenets associated with rigorous protocols [26], including multiple experts with diverse backgrounds, training of experts with the necessary vocabulary and concepts, following a structured elicitation protocol that privately recorded individual judgments before encouraging discussion among participants, and quantifying uncertainty around parameter estimates [30,82,83]. One limitation is the potential bias of the perspective of expertise by including veterinarians as the only profession represented, though they did come from diverse regional and personal backgrounds.

It may be perceived that the sample size here (number of participants) may be relatively small, compared to the population of field experts. The definition of sample size when consulting experts is subjective and, in many cases, a sample size of even one single expert has been used to parameterize distributions [84]; see also the discussion of sample size in [80]. Rather than numbers, we focused on giving our population the required training to help them understand what we wanted to estimate and then relied on their expertise and consensus-building to arrive at the best representation of each value. That said, results should be interpreted in light of the relatively few responses in Part B for the feedlot and ranching systems in Kenya and pastoral systems in Uganda. It is desirable to have several experts contributing knowledge because each tends to be overconfident in their own judgment (i.e., they specify bounds for a parameter that are too narrow), and the aggregation of uncertainty across several experts, as well as interaction and discussion among them, increases the consistency of expert knowledge with reality [28,80]. Because fewer individuals contributed to the aggregate distribution, there may be less uncertainty expressed for the parameter values than would have been covered with a greater number of contributors with expertise in these systems. Even so, the values of the estimates reported by our participants are generally supported: they are plausible compared to known values, supported by the consensus of the group, and align with trends shown in other literature.

Finally, the risk model structure and parameters were handled and influenced by the primary researcher and discussion facilitator, who is not from East Africa. This researcher built the preliminary model structure and questionnaires based on a literature review, reviewed and aggregated the individual results, facilitated the group discussion, and was involved in all decisions regarding data analysis and interpretation. The participants were invited to review and discuss the conclusions from each stage of the research process, including the report summarizing the process, final risk tree, and parameter distributions. It is possible that misinterpretation [80,85] could have occurred in both directions during communication between the researcher and the participants and is certain that the lens of the primary researcher has been incorporated into the final risk-mapping outputs. 

## 5. Conclusions

The results of this study fill the gap of identifying risk pathways and quantifying key variables for which published data are not available that are representative of the East African cattle management systems and value chains. This information could be combined with other available data to perform systematic risk assessment to estimate the baseline and relative risk for FMD transmission associated with beef products and to identify key variables for intervention including populations of focus, design of risk mitigation measures, and evaluation of what level of risk is reasonably achievable and at what cost. The novel approach builds on prior participatory and expert elicitation approaches to risk assessment to generate credible data appropriate for use in formal risk assessment models from local veterinary professionals.

## Data Availability

The data presented in this study are available in the article and supplementary material and available upon request.

## Supplementary Materials

The following are available online at https://www.mdpi.com/article/10.3390/v13112112/s1, File S1: Part A questionnaire, File S2: Part B questionnaire.

## Appendix A

**Table A1:** Group consensus and final distribution for each variable for cattle production systems in Kenya.

Variable Description	System	Consensus ^†^	Final Distribution	Distribution Median (5–95% Range)
Probability that cattle infected with FMD are sold for slaughter	Agropastoral	0.20 (0.1–0.3)	~PERT ()	0.2 (0.14–0.26)
	Pastoral			
	Ranching			
	Feedlot			
Days from sale/leaving the herd until slaughter	Agropastoral	3 (0.5–30)	~Gamma (1.8, 0.28)	5 (1–15)
	Pastoral	8 (0.5–21)	~Gamma (4.5, 0.5)	8 (3–17)
	Ranching	1 (0.5–3)	~Gamma (8.5, 6.8)	1.2 (0.6–2)
	Feedlot	1 (0.5–2)	~Gamma (15.1, 14.0)	1 (0.7–1.6)
Probability that cattle sold do not commingle with cattle from other herds	Agropastoral	0.1	0.1	NA
	Pastoral	0.05	0.05	NA
	Ranching	0.95	0.95	NA
	Feedlot	0.95	0.95	NA
Number of cattle mixed with when commingling does occur	Agropastoral	Individual estimates: median = 19; 90% range = 3–75	~Nbinom (1.2, 26.2)	19 (1–75)
	Pastoral			
	Ranching			
	Feedlot			
Probability that cattle bypass all inspection before slaughter	Agropastoral	0.2 (0.1–0.3)	~PERT ()	0.2 (0.14–0.26)
	Pastoral	0.4 (0.2–0.6)	~PERT ()	0.4 (0.28–0.52)
	Ranching	0.02 (0.01–0.05)	~PERT ()	0.02 (0.01–0.04)
	Feedlot	0.01 (0.01–0.05)	~PERT ()	0.02(0.01–0.03)
Number of inspections when cattle are inspected at least once	Agropastoral	1 (1–3)	({1,2,3}, {0.5, 0.33, 0.17})	1 (1–3)
	Pastoral			
	Ranching	2 (1–2)	({1,2}, {0.25, 0.75})	1 (1–2)
	Feedlot			
Effectiveness for type 1 inspection to detect and report/remove clinically infected cattle	Agropastoral	Individual estimates: median = 0.71 range = 0.15–1.0	~Beta (1.9, 0.8)	0.75 (0.23–0.99)
	Pastoral			
	Ranching			
	Feedlot			
Effectiveness for type 2 inspection to detect and report/remove clinically infected cattle	Agropastoral	Individual estimates: median = 0.56; range = 0.05–0.98	~Beta (1.6, 1.5)	0.52 (0.12–0.91)
	Pastoral			
	Ranching			
	Feedlot			
Relative frequency of each type of inspection	Agropastoral	Calculated from individual estimates	0.86, 0.14	NA
	Pastoral		0.66, 0.34	NA
	Ranching		1.0, 0	NA
	Feedlot		1.0, 0	NA

**^†^** Represents consensus from the group discussion unless otherwise indicated.

**Table A2:** Group consensus and final distribution for each variable for cattle production systems in Uganda.

Variable Description	System	Consensus ^†^	Final Distribution	Distribution Median (5–95% Range)
Probability that cattle infected with FMD are sold for slaughter	Agropastoral	0.3 (0.2–0.4)	~PERT ()	0.3 (0.24–0.36)
	Pastoral			
	Ranching			
	Semi-intensive			
Days from sale/leaving the herd until slaughter	Agropastoral	2 (0–7)	~Lognormal (0.84, 0.49)	2.3 (1–5)
	Pastoral			
	Ranching			
	Semi-intensive			
Probability that cattle sold do not commingle with cattle from other herds	Agropastoral	0.2 (0–0.5)	~ PERT ()	0.21 (0.07–0.38)
	Pastoral	0 (0–0)	~ PERT ()	0
	Ranching	0.4 (0.3–0.5)	~ PERT ()	0.4 (0.34–0.46)
	Semi-intensive	0.25 (0–0.7)	~ PERT ()	0.28 (0.08–0.51)
Number of cattle mixed with when commingling does occur	Agropastoral	15 (1–50)	~Nbinom (5.0, 18.4)	17 (6–35)
	Pastoral			
	Ranching			
	Semi-intensive			
Probability that cattle bypass all inspection before slaughter	Agropastoral	0.4 (0.35–0.45)	~PERT ()	0.4 (0.37–0.43)
	Pastoral	0.5 (0.4–0.6)	~PERT ()	0.5 (0.44, 0.56)
	Ranching	0.3 (0.25–0.35)	~PERT ()	0.3 (0.27–0.33)
	Semi-intensive	0.25 (0.1–0.4)	~PERT ()	0.25 (0.16, 0.34)
Number of inspections when cattle are inspected at least once	Agropastoral	1(1–3)	({1,2,3}, {0.5, 0.33, 0.17})	1 (1–3)
	Pastoral			
	Ranching			
	Semi-intensive			
Effectiveness for type 1 inspection to detect and report/remove clinically infected cattle	Agropastoral	Individual estimates: median = 0.83; range = 0.5–1.0	~Beta (8.9, 1.7)	0.86 (0.63–0.97)
	Pastoral			
	Ranching			
	Semi-intensive			
Effectiveness for type 2 inspection to detect and report/remove clinically infected cattle	Agropastoral	Individual estimates: median = 0.52; range = 0.2–0.9	~Beta (6.6, 5.7)	0.54 (0.31–0.76)
	Pastoral			
	Ranching			
	Semi-intensive			
Relative frequency of each type of inspection	Agropastoral	Calculated from individual estimates	0.48, 0.52	NA
	Pastoral		0.60, 0.40	NA
	Ranching		0.54, 0.46	NA
	Semi-intensive		0.53, 0.47	NA

**^†^** Represents consensus from the group discussion unless otherwise indicated.
