# Supplementary material for: Self-Reporting of Risk Pathways and Parameter Values for Foot-and-Mouth Disease in Slaughter Cattle from Alternative Production Systems by Kenyan and Ugandan Veterinarians
Source: Viruses. 2021 Oct 20;13(11):2112. doi: 10.3390/v13112112 (PMC8621966; doi:10.3390/v13112112)
Supplement: Supplementary file 1 [file viruses-13-02112-s001.zip › Supplementary Material File S1.pdf]

# Supplementary Materials File S1

Before diving into the modeling steps, it would be helpful to describe the systems we are talking about based on your experience.

Please answer the following questions. Your answers should range from 1-2 sentences to 1-2 paragraphs (depending on the breadth and depth of your insight into each question). You may have long answers to some questions and short or no answers to others—that is fine.

You may be familiar with various production systems, marketing chains, and/or regions that have diverse answers to the questions. If so, please describe each, and identify when/where each one takes place.

1. Describe the process and steps a bovine goes through when sold for meat:
    - From farm to slaughter
    - From slaughter to retail

2. What is the duration of the whole process, and of each step?

3. At what point/s are the cattle mixed with animals from other herds or regions? How many others, of what species, and for how long?

4. At what points in the process are animals examined for FMD? Formally and informally – e.g. including when examined by a trader or middleman. What happens if an animal is diagnosed or suspected to have FMD or any other disease?

5. Are there any points at which an animal with FMD (diagnosed or undiagnosed) would be more likely to be sold for meat than an animal without? If so, describe the circumstances.

6. Are any of these steps or the process influenced by (or differ by) season? This would include anything that is impacted by climate/weather, cultural events, or other market (supply and/or demand) dynamics.

7. When a case of FMD is suspected (and/or diagnosed), what changes occur in the sale process? How soon does that take place, what is the geography/population affected by those changes, and how long do the changes last?

## Model-building steps

Of course, our ProgRESSVet-Uganda question is not about oversleeping. Using the model-building steps, however, the ProgRESSVet team constructed a preliminary risk model to the question of FMD release through the supply chain of cattle and beef, and **now we will ask for your reflection and feedback on what we constructed**. This provides you an animal health example to learn from and, with your experience and expertise, an opportunity for us to learn and integrate information into our model. You will find questions throughout this section to focus your responses and to document your knowledge.

**Summary of risk assessment steps: Model Structure**

A. Clearly define the purpose of the risk assessment, the population of focus, and specific metrics that will be used to describe the event of interest.
B. Construct a fault tree.
C. Use the fault tree to construct an event tree (also called a scenario tree).
D. Label the variables on the event tree.
E. Use the event tree to describe the possible risk pathways
F. Summarize the variables, values needed, and potential information sources in a table.

## A. Clearly define the purpose of the risk assessment, the population of focus, and specific metrics that will be used to describe the event of interest.

The primary purpose or question that we are attempting to understand throughout this process is: what is the annual risk that beef or beef products produced in Uganda (and from subpopulations within Uganda) contain FMD virus?

To understand the broad question above, we will look at two questions more specifically:

- What is the risk that cattle sold for meat are carrying FMDV at slaughter?
- What is the risk that any kilogram of retail beef contains FMDV?

There are a number of specific ways of quantifying that risk, such as:

- Probability that a single bovine carries the virus at slaughter
- Expected number of affected cows slaughtered / year
- Annual probability of at least 1 affected cow slaughtered
- Number of years until the first event occurs
- *Equivalent metrics for a kilogram of retail beef*

We will focus here on the events between production and slaughter of a cow infected with FMDV, but the final model will also include processing steps in order to estimate the risk that any kilogram of beef produced is carrying FMDV.

## B. Construct a fault tree.

As you read above, a fault tree is a graphical depiction of the ways an event can occur. It starts with the event of interest on top and uses “And” and “Or” connectors to show how different events combine to arrive at the target event. Any of the alternative paths are sufficient to cause the top event.

The fault tree below, which the ProgRESSVet team created, explores the question: What are the possible combinations of events that can lead to a bovine carrying infectious FMDV at the time of slaughter?
This is a somewhat generic series of events, and you are probably thinking about specific markets and value chains with which you are familiar. The goal is to try to stay simple when possible (as described in

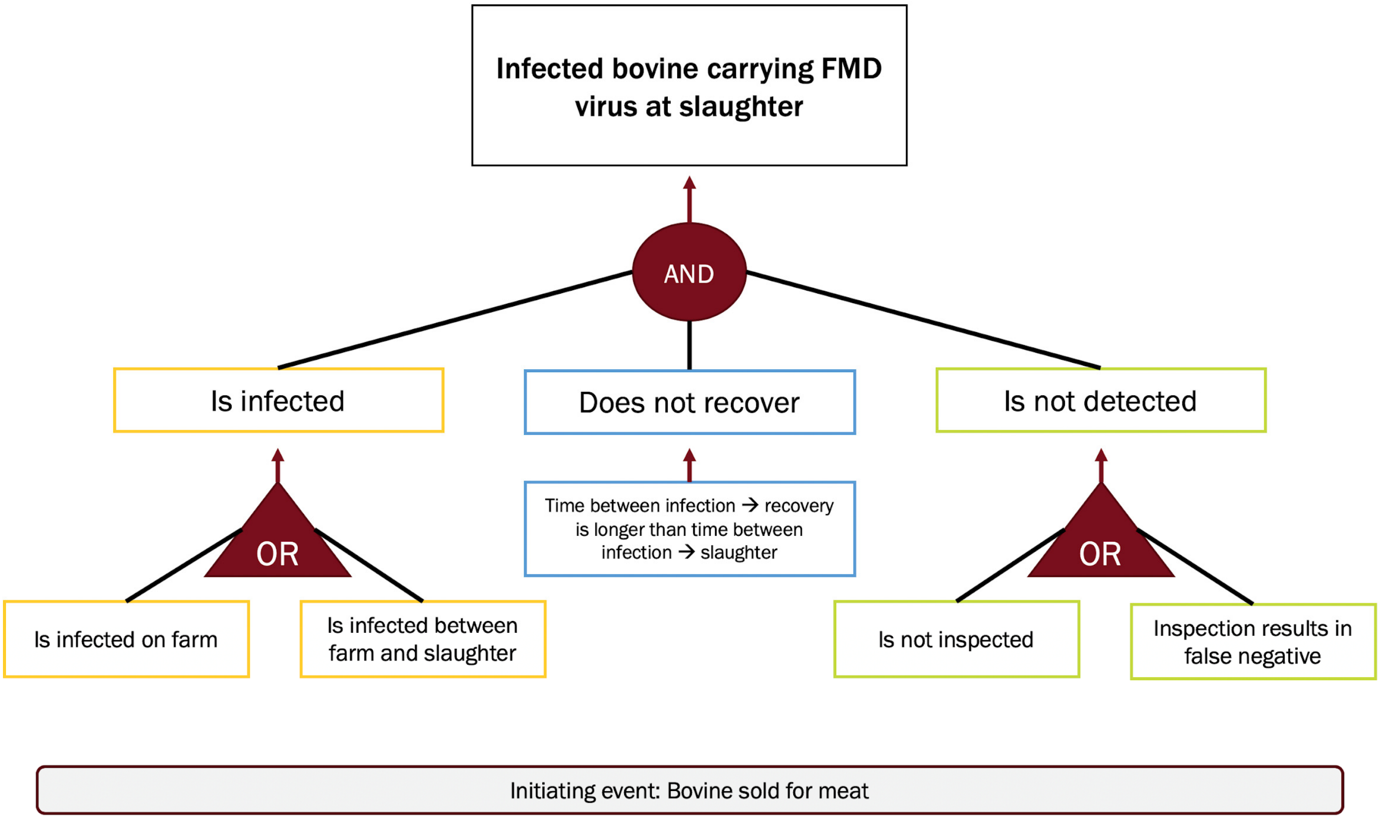


the OIE handbook, page 99!) but it is crucial that the simple representation captures all of the possible combinations of events we could care about.

8. Is there any example you can think of that does not fit (is not described by) these events?

## C. Use the fault tree to construct an event tree (also called a scenario tree).

As a reminder, an event tree is a graphical depiction of a sequence of events, progressing from an initiating event to an end state. The event tree helps us to organize the events represented in the fault tree into discrete pathways, and to identify the variables we need in order to quantify the frequency of those pathways. The events are placed across the top of the tree, and a branching line shows the various scenarios in which those events can be combined. The scenarios which represent the combinations in the fault tree are the pathways that result in our event of interest, so we will call them "risk pathways."

This event tree explores the question: What are the specific pathways that are individually sufficient to lead to the outcome of interest (a cow sold for meat carrying FMDV at slaughter)? As a reminder, we used the fault tree to create this event tree.

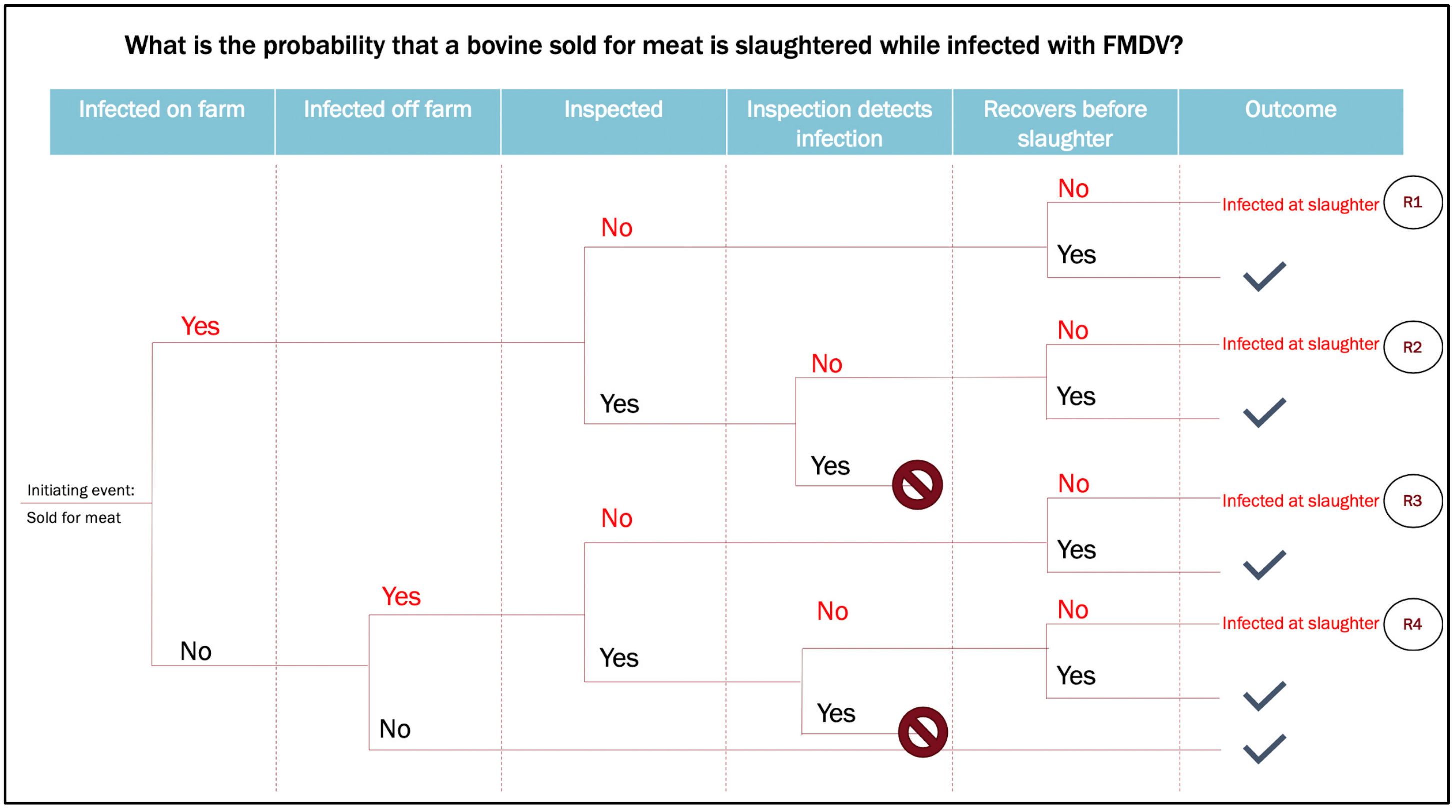


9. Do these pathways (R1 - R4) make sense to you? If not, what is confusing? Did you identify other potential pathways that could lead to an infected animal slaughtered?

## D. Label the variables on the event tree.

The event tree makes it evident which pieces of information we will need in order to answer our question. These key pieces of information are our variables, and we label them on the tree for easy reference. Since each variable is the probability that an event does/doesn't occur, we can represent it as Px for the "yes" and (1-Px) for the "no" options – the event either does or does not take place, and those two probabilities need to add up to 1.

Note that when the same event occurs on two different pathways, we name it as two different variables. This is because the nature of that event can look very different based on the events that have preceded it, so we want to consider it separately in each context. Even though it is the same event, it represents a distinct phenomenon on each path!

**What is the probability that a bovine sold for meat is slaughtered while infected with FMDV?**

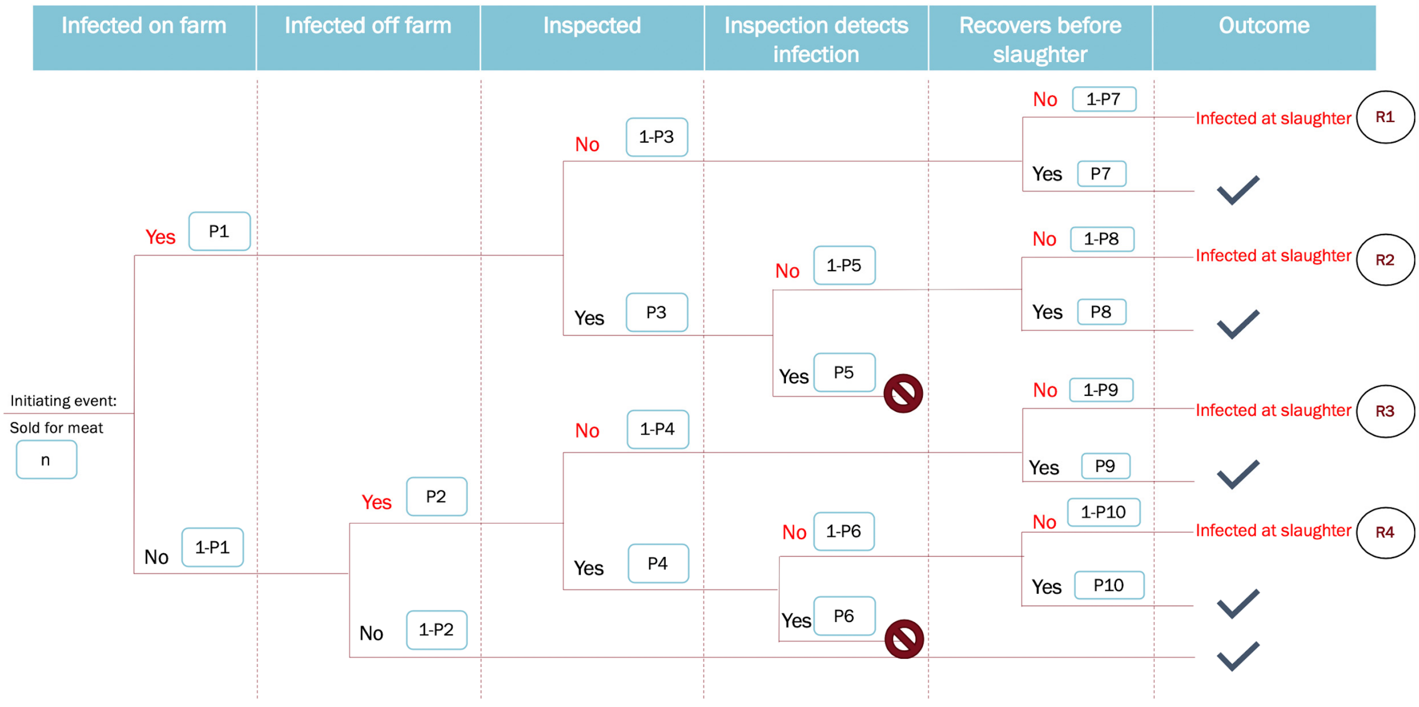


10. As you think about specific value chains you are familiar with, are there additional variables that you consider important that you do not see written here? What are they, and how are they related to the variables shown?

## E. Use the event tree to describe the possible risk pathways

By using the logic of the tree we have built and basic rules of probability, we can now represent the risk pathways in terms of the variables in each.

| Risk pathway | Description | Inputs |
|---|---|---|
| R1 | Infected on farm, not inspected, does not recover | P1*(1-P3)*(1-P7) |
| R2 | Infected on farm, inspected with false negative result, does not recover | P1*P3*(1-P5)*(1-P8) |
| R3 | Infected off farm, not inspected, does not recover | (1-P1)*P2*(1-P4)*(1-P9) |
| R4 | Infected off farm, inspected with false negative result, does not recover | (1-P1)*P2*P4*(1-P6)*(1-P10) |

Risk that a single animal sold for meat is infected at the time of slaughter = the sum of the probabilities that he/she experienced any one of those 4 individually-sufficient pathways = R1 + R2 + R3 + R4

*** Differentiate between subpopulations**

If we thought that we had a homogenous source population, then we could skip to the variable summary (last step). But since we suspect that the values for some variables will differ according to the population to which an animal belongs, we want to differentiate between those distinct subpopulations.

One option is to define subpopulations based on Production System, specifically as described by the FAO in Africa Sustainable Livestock 2050: Cattle and poultry sector in Uganda (page 2)

| **Table A1.** Cattle (beef) production in Uganda | |
|---|---|
| **Ranching** | In ranching systems, farmers keep large numbers of animals (500 - 3,000 per holding) in perimeter fencing, paddocked structures and grazing fields. They keep a mixture of indigenous, cross and exotic beef animals and make substantial investment in animal health management, the purpose of ranching systems being the production and marketing of beef, with milk being a by-product. This system is prevalent in the Southwest and the Central 2 sub-regions. |
| **Pastoral** | In pastoral or free grazing systems, farmers move cattle from place to place in search of pastures and water. They keep indigenous breeds, with herd size ranging from few to 100 heads. Main products include beef, milk, blood, hides, manure and horns. This system is dominant in the Northeastern sub-region. |
| **Agro Pastoral** | Farmers graze largely indigenous cattle in both private and public pastures and also feed them with crops by-products. Cattle produce beef and milk, hides, manure and horns and also provide draught power. Investments in improved husbandry practices, including animal health, are none to minimal. This system is present in the Eastern, Central 2, Western, North and West Nile Sub-regions. |
| **Semi-intensive** | Farmers keep cattle, mainly cross-bred, confined in kraals, paddocks and cattle barns/stalls and feed them with compound feed. They also make significant investments in animal health, such as in vaccination and deworming. Cattle produce milk and beef. This system is mainly found in Central 1 and 2 and the Southwest sub-regions. |
| *Source: FAO ASL2050 (2017), based on National expert consultation* | |

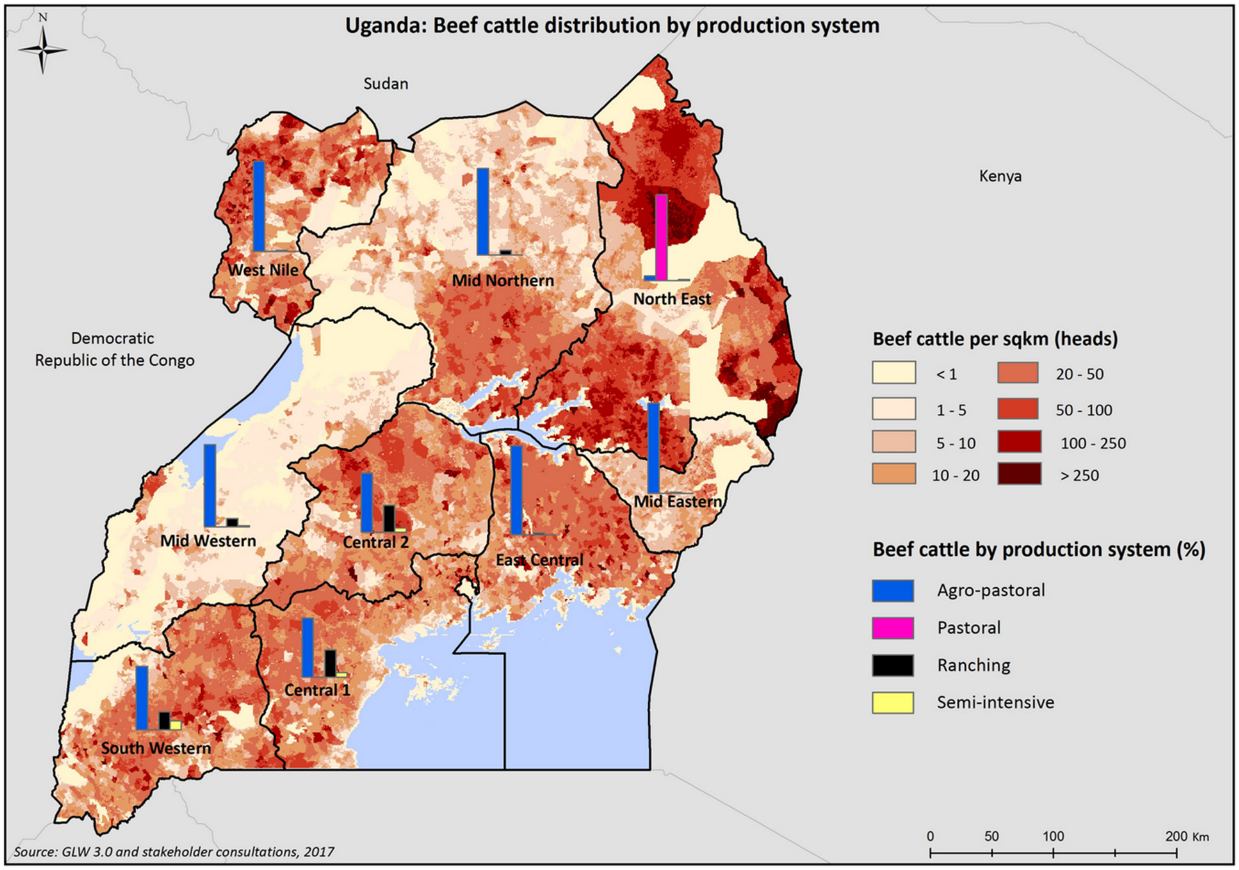


Source: Africa Sustainable Livestock 2050

**Do we expect each variable to differ based on the subpopulation to which it belongs?**
**If yes, which factors would you expect to influence that difference?** These factors may be differences during production or differences in what the animal experiences after it has left the farm.

For example: P1 = probability that a cow sold for meat is infected while still on the farm. I may think that the risk of infection is different for an animal in a pastoralist system than in a ranching system, because of differences due to the region (pastoralist herds tend to be close to international borders where the risk is higher) and due to management (pastoralist herds tend to move and co-mingle with other populations, increasing the risk of acquiring infection).

11. Please indicate in the table below which variables you expect to differ between these 4 subpopulations (agro-pastoral, pastoral, ranching, and semi-intensive production systems, as described above). Write yes or no in the third column, and then explain your reasoning in the space on the next page.

*Note: we are not asking you what these values are, just to think about whether those values would be different for animals coming from different populations.*

| **Variable** | **Description** | ***Do you expect this value to be different for animals that belong to different source populations? (Y/N)*** |
|---|---|---|
| P1 | Probability that a cow sold for meat **is infected while on farm** | |
| P2 | Probability that a cow sold for meat **is infected between farm and slaughter** | |
| P3 | Probability that a cow sold for meat and infected on farm **is inspected for FMD before slaughter** | |
| P4 | Probability that a cow sold for meat and infected between farm and slaughter **is inspected for FMD before slaughter** | |
| P5 | Probability that a cow sold for meat, infected on farm, and inspected for FMD **is identified as infected with FMD** | |
| P6 | Probability that a cow sold for meat, infected between farm and slaughter, and inspected for FMD **is identified as infected with FMD** | |
| P7 | Probability that a cow sold for meat, infected on farm, and not inspected for FMD **recovers from infection before slaughter** | |
| P8 | Probability that a cow sold for meat, infected on farm, inspected for FMD with a false negative result **recovers from infection before slaughter** | |
| P9 | Probability that a cow sold for meat, infected between farm and slaughter, and not inspected for FMD **recovers from infection before slaughter** | |
| P10 | Probability that a cow sold for meat, infected between farm and slaughter, inspected for FMD with a false negative result **recovers from infection before slaughter** | |
| n | Number of bovines sold for meat per year | |

12. For each of the variables above that you answered “Yes,” please explain your reasoning. Which populations would you expect to be different from one another and what would be the reason for those differences? If you identified additional variables above, please answer the same questions for them.

13. Would you recommend a different way of dividing and identifying subpopulations?

### F. Summarize the variables, values needed, and potential information sources in a table.

Finally, we want to organize all of the information we will need and identify potential sources of data that can be used to describe the input variables.

14. Please read the descriptions of each of the input variables listed in the table below. In the "source" column, please note if you have any ideas of sources of information relevant to that particular variable. The information source could be a ministry or project report, data on health events from your district, or a person who you think would have expertise in that area. This could be data specific to one of the subpopulations identified above, or general to all of Uganda.

*You do not need to understand all of the equations and relationships between variables, though the logic should be straightforward. If you have questions, we would be happy to discuss anything you find unclear.*

| **Variable*** | **Description** | **Input** | ***Source*** |
|---|---|---|---|
| **P1_j** | Probability that a cow sold for meat **is infected with FMD** on a given day while on farm | cases*duration / (population*365) | |
| cases_j | Number of FMD cases per year | --- | |
| duration | Duration of FMD infection (days) | --- | |
| population_j | Number of cattle eligible to be sold for meat | --- | |
| | | | |
| **P2_j** | Probability that a cow sold for meat **is infected between farm and slaughter** | Pmix*mixprev*Ptransmission | |
| Pmix_j | Probability that a cow in each subsystem is commingled with animals from other herds between farm and slaughter | --- | |
| mixprev | Probability that at least 1 animal is infected among those commingled with | 1-(1-P1)^{nmix} | |
| nmix | Number of animals commingled with | --- | |
| Ptransmission | Probability that commingling with at least 1 infected animal results in infection | --- | |
| | | | |

| | | | |
|---|---|---|---|
| **P3_j, P4_j** | Probability that a cow sold for meat and infected (on farm, between farm and slaughter) **is inspected for FMD before slaughter** | --- | |
| | | | |
| **P5_j, P6_j** | Probability that a cow sold for meat, infected (on farm, between farm and slaughter) and inspected for FMD **is identified as infected with FMD** | se1_j, se2_j | |
| se1_j, se2_j | Sensitivity of inspection/test to detect FMD infection among animals from population j (who were infected on farm, who were infected between farm and slaughter) | # identified as positive / # infected and inspected | |
| | | | |
| **P7_j - P10_j** | Probability that a cow sold for meat… **recovers from infection before slaughter** | P(duration > lag_farm), P(duration > lag_off-farm) | |
| lag_farm | Days between sale from farm and slaughter | --- | |
| lag_off-farm | Days between infection and slaughter | --- | |
| | | | |
| n_j | Number of bovines sold for meat per year | --- | |

*The subscript j indicates that there is a unique version of that variable for each of the 4 subpopulations. For example, there is a P1_AgroPastoral, P1_Pastoral, P1_Ranching, and P1_Semi-intensive.

## Final considerations

15. Please review your answers from the beginning of the exercise. Does anything new come to mind after working through these modeling steps?

16. What are the **control measures** which are deployed to mitigate the risk of FMD? How do they differ by each subpopulation? That may include:
    - Movement controls / quarantines (regardless of infection status)
    - Inspections (goal = detection and then action taken re: the infected individuals or herds)

17. Please describe the ways that **the actions and responsibilities of veterinary services** impact the probabilities and events that contribute to risk as discussed here. This could be:
    a. the risk of FMD in cattle herds/animals
    b. the risk of the meat from an FMD-infected animal making it into the food supply

18. What are ways that the **structure, organization, and/or approach** of veterinary services in Uganda contributes to effectively controlling and preventing these events from occurring? What are ways that VS structure and function could more effectively contribute to this goal? Consider the roles, relationships, and efficacy of central/field, public/private, and vet/paravet/animal health worker dynamics.

19. Do you suggest any resources or references that would provide additional information? This could include written or human sources.
